# Identification and Functional Characterization of a Highly Divergent *N*-Acetylglucosaminyltransferase I (TbGnTI) in *Trypanosoma brucei*

**DOI:** 10.1074/jbc.M114.555029

**Published:** 2014-02-18

**Authors:** Manuela Damerow, Joao A. Rodrigues, Di Wu, M. Lucia S. Güther, Angela Mehlert, Michael A. J. Ferguson

**Affiliations:** From the ‡ Division of Biological Chemistry and Drug Discovery, College of Life Sciences, University of Dundee, Dundee DD1 5EH, United Kingdom and; the § Instituto de Medicina Molecular, Faculdade de Medicina da Universidade de Lisboa, 1649-028 Lisboa, Portugal

**Keywords:** Glycobiology, Glycosyltransferases, Parasite, Post-translational Modification, Trypanosoma brucei, N-Acetylglucosamine

## Abstract

*Trypanosoma brucei* expresses a diverse repertoire of *N*-glycans, ranging from oligomannose and paucimannose structures to exceptionally large complex *N*-glycans. Despite the presence of the latter, no obvious homologues of known β1–4-galactosyltransferase or β1–2- or β1–6-*N*-acetylglucosaminyltransferase genes have been found in the parasite genome. However, we previously reported a family of putative UDP-sugar-dependent glycosyltransferases with similarity to the mammalian β1–3-glycosyltransferase family. Here we characterize one of these genes, *TbGT11*, and show that it encodes a Golgi apparatus resident UDP-GlcNAc:α3-d-mannoside β1–2-*N*-acetylglucosaminyltransferase I activity (TbGnTI). The bloodstream-form *TbGT11* null mutant exhibited significantly modified protein *N*-glycans but normal growth *in vitro* and infectivity to rodents. In contrast to multicellular organisms, where the GnTI reaction is essential for biosynthesis of both complex and hybrid *N*-glycans, *T. brucei TbGT11* null mutants expressed atypical “pseudohybrid” glycans, indicating that TbGnTII activity is not dependent on prior TbGnTI action. Using a functional *in vitro* assay, we showed that TbGnTI transfers UDP-GlcNAc to biantennary Man_3_GlcNAc_2_, but not to triantennary Man_5_GlcNAc_2_, which is the preferred substrate for metazoan GnTIs. Sequence alignment reveals that the *T. brucei* enzyme is far removed from the metazoan GnTI family and suggests that the parasite has adapted the β3-glycosyltransferase family to catalyze β1–2 linkages.

## Teaser

**Background:** *Trypanosoma brucei* expresses a highly glycosylated surface coat that is essential for parasite survival.

**Results:** The *T. brucei* gene *TbGT11* encodes an *N*-acetylglucosaminyltransferase I, the key enzyme for initiating the biosynthesis of complex *N*-glycans.

**Conclusion:** *T. brucei* GnTI is not a homologue of metazoan GnTI, but a highly divergent enzyme belonging to the β3-glycosyltransferase family.

**Significance:** Deeper understanding of *T. brucei N*-glycosylation pathway.

## Introduction

African trypanosomes are tsetse fly-transmitted protozoan parasites that cause human African sleeping sickness and nagana in livestock. *Trypanosoma brucei* undergoes a complex life cycle, adapting to a bloodstream form in the mammalian host, where it lives and divides extracellularly in the blood, lymph, and interstitial fluids. A densely packed layer of glycosylphosphatidylinositol (GPI)^6^-anchored variant surface glycoprotein (VSG) covers the parasite cell surface (1). Apart from serving as a physical barrier to components of the host complement system, this VSG coat undergoes antigenic variation that allows the parasite to persist in the host bloodstream (2, 3). The cell line used in this study (strain 427) expresses VSG221, which contains a galactosylated GPI anchor (4) and two types of *N*-glycans; triantennary oligomannose structures (Man_7–9_GlcNAc_2_) at Asn-428 and biantennary paucimannose (Man_3–4_GlcNAc_2_) and small complex (Gal_0–2_GlcNAc_1–2_Man_3_GlcNAc_2_) structures at Asn-296 (5).

*N*-Glycan biosynthesis takes place in the endoplasmic reticulum (ER) and Golgi apparatus as a non-template assembly line. The precursor for *N*-glycans is built on the lipid carrier dolichol pyrophosphate (Dol-PP) in the ER membrane and, in higher eukaryotes, ends in the formation of Glc_3_Man_9_GlcNAc_2_-PP-Dol. The action of an oligosaccharyltransferase transfers the glycan portion *en bloc* to the nascent glycoproteins. Subsequent processing reactions trim Glc_3_Man_9_GlcNAc_2_ down to a triantennary Man_5_GlcNAc_2_ structure (6). The first step in hybrid and complex *N*-glycan biosynthesis is initiated by *N*-acetylglucosaminyltransferase I (GnTI) through the addition of an *N*-acetylglucosamine (GlcNAc) residue to the α1-3-linked core mannose of Man_5_GlcNAc_2_. In multicellular organisms, the GnTI reaction generally precedes the subsequent trimming reactions by Golgi α-mannosidase II (7, 8) and is a prerequisite for GlcNAc transfer to the α1-6-linked Man of GlcNAcMan_3_GlcNAc_2_ by *N*-acetylglucosaminyltransferase II (GnTII) (9, 10). The absence of GnTI (and therefore a complete deficiency of complex *N*-glycans) has been shown to be embryonic lethal in mice (11, 12).

In contrast, *T. brucei* expresses two oligosaccharyltransferases with different substrate and acceptor specificities, one (TbSTT3A) that transfers biantennary Man_5_GlcNAc_2_ to relatively acidic glycosylation sites (*e.g.* Asn-263 of VSG221) and another (TbSTT3B) that transfers Man_9_GlcNAc_2_ to any remaining sites (*e.g.* Asn-428 of VSG221) (13–16). It is proposed that for the generation of complex *N*-glycans in *T. brucei,* Man_5_GlcNAc_2_ is processed down to Man_3_GlcNAc_2_ and that this serves as a substrate for both *T. brucei* TbGnTI and TbGnTII. Thus, the actions of these two enzymes are suggested to be independent of each other, which would imply that the GlcNAc transferases involved in complex *N*-glycan biosynthesis in *T. brucei* may be different from their metazoan counterparts (15–18). Indeed, no obvious GnTI or GnTII homologs have been identified in the parasite genome (19) and, so far, only GPI anchor (20, 21) and unspecified GlcNAc transferase activities (22, 23) have been detected using *T. brucei* cell-free systems.

A minimum of 38 distinct glycosidic linkages have been identified in the *T. brucei* glycome (19), however, so far only six glycosyltransferases have been experimentally related to specific genes: UDP-Glc:glycoprotein α1-3-glycosyltransferase to *TbUGGT* (24), dolichol phosphate mannose synthase to *TbDPMS* (25), Dol-P-Man:Man_5_GlcNAc_2_ α1–3-mannosyltransferase to *TbALG3* (16), Dol-P-Man:Man_7_GlcNAc_2_ α1–6-mannosyltransferase to *TbALG12* (17, 18), Dol-P-Man:Man_2_GlcNPI α1-2-mannosyltransferase to *TbGPI10* (26), and UDP-GlcNAc:β-d-Gal-GPI β1-3-GlcNAc transferase to *TbGT8* (19). In addition to these, another seven TbGT genes can be reasonably confidently assigned by sequence homology (*i.e. TbALG1*, *-2*, *-7*, -*9*, and -*11* and *TbGPI14* and -*18*). However, that still leaves a minimum of 25 glycosidic linkages looking for requisite GT genes. Intriguingly, searches of the *T. brucei* genome using a human β1-3-*N*-acetylglucosaminyltransferase sequence (β3GnT5) as the query revealed 21 full-length ORFs encoding putative UDP-Gal or UDP-GlcNAc-dependent GTs, only one of which (TbGT8) has been characterized to date (19).

In the present study, we analyzed another of these putative UDP-sugar-dependent GTs by a reverse genetics approach and by *in vitro* functional activity assay. Our study revealed that the gene *TbGT11* (Tb427.3.5660) encodes a UDP-GlcNAc:α3-d-mannoside β1-2-*N*-acetylglycosaminyltransferase I activity (EC 2.4.1.101) involved in the biosynthesis of complex *N*-glycans and revealed significant differences between the parasite enzyme and its metazoan counterparts both in amino acid sequence and substrate specificity.

## EXPERIMENTAL PROCEDURES

### 

#### 

##### Cultivation of Trypanosomes

*T. brucei brucei* strain 427 bloodstream-form parasites, expressing VSG variant 221 and transformed to stably express T7 polymerase and the tetracycline repressor protein under G418 antibiotic selection (27), were used in this study. This genetic background will be referred to hereon as wild-type (WT). Cells were cultivated in HMI-9 medium containing 2.5 μg/ml of G418 at 37 °C in a 5% CO_2_ incubator as described in Ref. 27.

##### DNA and RNA Isolation and Manipulation

Plasmid DNA was purified from *Escherichia coli* (α-select chemically competent cells, Bioline, London, UK) using Qiagen Miniprep or Maxiprep kits, as appropriate. Gel extraction and reaction cleanup was performed using Qiaquick kits (Qiagen). Custom oligonucleotides were obtained from Eurofins MWG Operon or the Dundee University oligonucleotide facility. *T. brucei* genomic DNA was isolated from ∼2 × 10^8^ bloodstream-form cells using DNAzol (Helena Biosciences, UK) using standard methods. *T. brucei* mRNA was extracted from 1 × 10^7^ cells using RNeasy RNA extraction kit (Qiagen).

##### Generation of Gene Replacement Constructs

The 554-bp 5′ and 584-bp 3′ UTR sequences next to the Tb427.3.5660 ORF were PCR amplified from genomic DNA using *Pfu* DNA polymerase with primers 5′-atatgtttGCGGCCGCgtgataatgttcatgcaatg-3′ and 5′-*gtttaaac*ttacggaccgtcaagctttggatgggtattacaaaaac-3′, 5′-gacggtccgtaagtttaaacggatcccttaagtgcaacaataacttt-3′ and 5′-tctgGTCGACgtagtgaacaaactgttagc-3′ as forward and reverse primers, respectively. The two PCR products were used together in a further PCR to yield a product containing the 5′-UTR linked to the 3′-UTR by a short HindIII, PmeI, and BamHI cloning site (underlined) and NotI and SalI restriction sites at the 5′ and 3′ end, respectively (capital letters). The product was cloned into the NotI site of the pGEM-5Zf(+) vector (Promega). An extra endogenous HindIII site identified in the 5′-UTR (AAGCTT) was replaced by (AAGTTT) using QuikChange Site-directed Mutagenesis Kits (Stratagene) according to the manufacturer's instruction with primers 5′-CCTTTTCTGTTCTATAGTTAAGTTTCATTGATAATCTAAACAAAC-3′ and 5′-CAAACAAATCTAATAGTTACTTTGAATTGATATCTTGTCTTTTCC-3′ as forward and reverse primers, respectively.

The hygromycin phosphotransferase (*HYG*) and puromycin acetyltransferase (*PAC*) drug-resistance genes were then introduced into the targeting vector via the HindIII and BamHI cloning sites. For re-expression of Tb427.3.5660 the ORF was PCR-amplified from genomic DNA with the primer pair 5′-gctGGATCCtatggttattcgctctcccg-3′ and 5′-aacCTCGAGaacgtagctatggggtgacg-3′ and cloned into pLEW100-Phleo (27).

For overexpression of full-length TbGT11 with a C-terminal HA_3_ epitope tag, a plasmid was generated based on the trypanosome expression vector pLEW82 (27). *TbGT11* ORF was amplified from *T. brucei* genomic DNA and primers 5′-GACTAAGCTTATGGCAATCAAATCACGAGGAG-3′ and 5′-GACTTTAATTAA*tgcgtaatcagggacgtcataaggatatgcgtaatcagggacgtcataaggata*cgctcccgcTGCCGTCCATTCCGCATCC-3′ containing a HindIII and PacI restriction site (underlined), respectively. The sequence encoding for two HA tags (italics) followed by a sequence encoding an Ala-Gly-Ala linker was attached as a 5′ overhang of the reverse primer. The PCR product was subcloned into pLEW82-*GPIdeAc-HA* (28) via HindIII and PacI restriction sites under replacement of the *GPIdeAc* insert, but retention of the sequence encoding for one HA tag, resulted in plasmid pLEW82-*TbGT11-HA_3_*. The identity of all constructs was confirmed by sequencing.

##### Transformation of Bloodstream-form T. brucei

Constructs for gene replacement and ectopic expression were purified, digested with NotI to linearize, precipitated, washed with 70% ethanol, and re-dissolved in sterile water. The linearized DNA was electroporated into *T. brucei* bloodstream-form cells (strain 427, variant 221) that were stably transformed to express T7 RNA polymerase and the tetracycline repressor protein under G418 selection. Cell culture and transformation were carried out as described previously (27–29).

##### Southern Blotting

Aliquots of genomic DNA isolated from 100 ml of bloodstream-form *T. brucei* cultures (∼2 × 10^8^ cells) were digested with ApaI, resolved on a 0.8% agarose gel and transferred onto a Hybond-N positively charged membrane (GE Healthcare, Amersham Biosciences). Highly-sensitive DNA probes labeled with digoxigenin-dUTP were generated using the PCR DIG Probe Synthesis Kit (Roche Applied Science) according to the manufacturer's recommendations and hybridized overnight at 42 °C. Detection was performed using alkaline phosphatase-conjugated anti-digoxigenin Fab fragments and the chemiluminescent substrate CSPD (Roche).

##### Mouse Infectivity Studies

Wild-type and *TbGT11* null mutant bloodstream-form trypanosomes were grown in HMI-9T media, washed in media without antibiotics and resuspended at 1 × 10^6^ cells/ml. Groups of 5 female Balb/c mice were used for each cell line and 0.1 ml of the suspension above was injected intraperitoneally per animal. Infections were assessed daily by tail bleeding and cell counting using a Neubauer chamber in a phase-contrast microscope.

##### Semi-quantitative RT-PCR

To assess the amount of Tb427.3.5660 mRNA in the *TbGT11* conditional null mutant cells grown under permissive and non-permissive conditions, RT-PCRs were performed using the AccessQuick RT-PCR System (Promega) according to the manufacturer's recommendations. A *TbGT11* 1074-bp fragment was amplified with the primer pair 5′-cgtttccaccaaaattccc-3′ and 5′-ttatgccgtccattccgcatc-3′. As a control of a similar RNA levels in both samples, primers 5′-aatggatgcggaccttcagcacccac-3′ and 5′-tagaaccgtgagcgcggtgccatac-3′ amplifying a 448-bp product of dolichol phosphate mannose synthase (Tb10.70.2610) were used.

##### Small Scale sVSG Isolation

Soluble-form VSG (sVSG) was isolated from 100 ml of cultures containing ∼2 × 10^8^ bloodstream-form *T. brucei* by a modification of the method of Cross (30, 31) as described in Ref. 17. Briefly, cells were chilled on ice, centrifuged at 2500 × *g* for 10 min, and washed in an isotonic buffer. The pellet was resuspended in 300 μl of lysis buffer (10 mm NaH_2_PO_4_ buffer, pH 8.0, containing 0.1 mm tosyllysine chloromethyl ketone hydrochloride (TLCK), 1 μg/ml of leupeptin, and 1 μg/ml of aprotinin) and incubated for 5 min at 37 °C. The sample was centrifuged at 14,000 × *g* for 5 min, and the supernatant was applied to a 200-μl DE52 anion exchange column pre-equilibrated in 10 mm sodium phosphate buffer, pH 8.0. Elution was performed with 0.8 ml of 10 mm sodium phosphate buffer, pH 8.0, the eluate was concentrated and diafiltered with water on a YM-10 spin concentrator (Microcon). The final sample of 50–100 μg of sVSG221 was recovered in a volume of 100 μl of water.

##### ES-MS Analysis of Intact sVSG

50-μg Aliquots of sVSG preparations were diluted to ∼0.05 μg/μl in 50% methanol, 1% formic acid and analyzed by positive ion ES-MS on a Q-TOF 6520 instrument (Agilent). Data were collected, averaged, and processed using the maximum entropy algorithm of the MassHunter software (Agilent).

##### Purification and MALDI-TOF Analysis of VSG GPI Anchors

Aliquots of purified sVSGs were treated with 50 μl of ice-cold 50% aqueous hydrogen fluoride for 48 h at 0 °C to cleave the GPI anchor ethanolamine-phosphate bond. The resultant GPI glycans were dried, re-dissolved in 50 μl of water, and transferred into a 2-ml Reactivial (Pierce). Samples were dried and then re-dried from 50 μl of methanol, followed by permethylation as described previously (32). Finally, permethylated glycans were mixed with 2,5-dihydroxybenzoic acid matrix and analyzed in positive-ion mode using an ABI Voyager DE-STR MALDI-TOF mass spectrometer.

##### ES-MS/MS Analysis of Pronase Glycopeptides

50-μg Aliquots of sVSG preparations were mixed with 5 μl of 1 m ammonium bicarbonate buffer and 10 μl of 1 mg/ml of freshly prepared Pronase (Sigma) dissolved in 5 mm calcium acetate and incubated for 48 h at 37 °C. In some experiments, the parasites were grown for 72 h in the presence of a mixture of α-mannosidase inhibitors (0.8 mm 1-deoxymannojirimycin (R&D Systems, Inc., Minneapolis, MN), 186 μm kifunensine (Santa Cruz Biotechnology, Dallas, TX), and 100 μm swainsonine (Sigma)) before sVSG isolation. The Pronase glycopeptides were purified using Envicarb graphitized carbon microcolumns as described previously (16, 33). Aliquots of these enriched glycan samples were loaded into nanotips (Waters-type F) and analyzed by ES-MS and ES-MS/MS in positive-ion mode on an LTQ Orbitrap XL mass spectrometer (Thermo Scientific) with tip and 1100 V. The product ion spectra of selected ions were collected using collision energies of 8–20 V. The ES-MS spectra were processed using the Thermo Xcalibur software.

##### Analysis of Total N-Glycans by Lectin Blotting and LC-MS

To analyze the total *N*-glycan fraction of *T. brucei* bloodstream-form cells, ∼2 × 10^9^ cells were first depleted of VSG by hypotonic lysis (30, 31). For Western blot analysis, residual cell ghosts were solubilized in a SDS sample buffer containing 8 m urea, boiled with DTT, separated by SDS-PAGE (approximately 1 × 10^7^ cell equivalents/lane) on NuPAGE bis-Tris 4–12% gradient acrylamide gels (Invitrogen), and transferred to nitrocellulose membrane (Invitrogen). Ponceau S staining confirmed equal loading and transfer. Glycoproteins were probed with 1.7 μg/ml of biotin-conjugated ricin (RCA-120, Vector Laboratories, Peterborough, UK) in PBS before or after preincubation with 10 mg/ml of d-galactose and 10 mg/ml of α-lactose to confirm specific ricin binding. Detection was performed using IRDye 680LT-conjugated streptavidin and the LI-COR Odyssey Infrared Imaging System (LICOR Biosciences, Lincoln, NE). For mass spectrometry analysis, glycans were purified by modification of the filter-aided sample preparation procedure (34). Briefly, cell ghosts were solubilized in 1 ml of 8% SDS, 200 mm DTT, 200 mm Tris-HCl, pH 8.0, per 1 × 10^9^ cell equivalents by vigorous vortexing for 3 min, sonication for 3 min, heating to 95 °C for 3 min, and a further 3-min vortexing step. After clearing the lysate by centrifugation at 16,000 × *g* for 5 min, the solubilized sample was reductively alkylated in a 30,000 molecular weight cutoff spin filtration unit (Sartorius AG, Goettingen, Germany) using the filter-aided sample preparation procedure II procedure adapted for larger volumes. Digest with 25 μg/ml of trypsin gold (Promega) was performed in the filtration unit overnight at 37 °C and peptides were eluted by centrifugation, whereas the bulkier glycopeptides remained in the retentate. Residual trypsin in the filter was inactivated by 50 μg/ml of trypsin inhibitor from bovine pancreas (Sigma), followed by two wash steps with 50 mm NH_4_HCO_3_. Glycopeptides were transferred into a microcentrifuge tube and incubated with 4 units of peptide:*N*-glycosidase F (Roche Applied Science) overnight at 37 °C. Finally, released glycans were desalted on a mixed-bed ion exchange column of 100 μl of Chelex-100 (Na^+^) over 100 μl of AG50X12 (H^+^), over 200 μl of AG3X4 (OH^−^), and over 100 μl of QAE-Sephadex25 (OH^−^), all from Bio-Rad Laboratories, except QAE-Sephadex (Sigma). After freeze-drying, glycans were permethylated as described previously (32) and dissolved in 10% methanol, 1% formic acid. Analysis was performed by LC-MS using a C18 reversed-phase column (Acclaim RSLC PepMap, 75 μm × 15 cm, Thermo Scientific) and a 10–90% acetonitrile gradient with a LTQ Orbitrap XL mass spectrometer (Thermo Scientific). All runs were done at a flow-rate of 0.3 μl/min and a column temperature of 30 °C. Extracted ion chromatograms were created using the Thermo Xcalibur software.

##### GnTI in Vitro Activity Assay

TbGT11 fused to a C-terminal triple HA tag was overexpressed in *T. brucei* bloodstream-form cells. 1 × 10^9^ cells were lysed on ice in 25 mm Tris, pH 7.5, 100 mm NaCl, 1% Triton X-100 containing a mixture of protease inhibitors (CompleteMini, Roche) and 0.1 mm TLCK. Expression was confirmed by SDS-PAGE and Western blotting. Briefly, 5 × 10^6^ cell equivalents/lane were separated on NuPAGE bis-Tris 4–12% gradient acrylamide gels (Invitrogen) and transferred to nitrocellulose membrane (Invitrogen). Ponceau S staining confirmed equal loading and transfer. Detection was performed using 0.5 μg/ml of rabbit anti-HA antibody (QED Bioscience Inc., San Diego, CA) and IRDye 680LT-conjugated donkey anti-rabbit IgG and the LI-COR Odyssey Infrared Imaging System (LICOR Biosciences). For the *in vitro* activity assay, TbGT11-HA_3_ was immunoprecipitated using anti-HA bead magnetic beads (Pierce) and incubated with 1 μCi of UDP-[^3^H]GlcNAc (specific activity of 20–40 Ci/mmol, PerkinElmer Life Sciences), 1 mm cold UDP-GlcNAc (Sigma), 5 μg of oligomannose-3 (a), or 5 μg of oligomannose-5 (Dextra Laboratories, Reading, UK) in 50 mm Tris, pH 7.5, 10 mm MgCl_2_, 10 mm MnCl_2_ in a total volume of 50 μl. After overnight incubation under vigorous shaking at room temperature, samples were desalted via a mixed-bed ion exchange column as described above, freeze-dried, and re-dissolved in 20% 1-propanol. Aliquots were spotted onto silica HPTLC plates (SI-60 HPTLC, Millipore) that were run twice in 1-propanol/acetone/H_2_O (9:6:4). For product analysis, samples were treated with α1–2,3-mannosidase from *Xanthomonas manihotis* (New England Biolabs) before TLC analysis. Plates were then dried, sprayed with EN^3^HANCE autofluorography enhancer (EN^3^HANCE, PerkinElmer Life Sciences), and exposed on x-ray film at −80 °C for 1–2 days.

##### Immunofluorescence Analysis

Bloodstream-form cells transformed with pLEW82-TbGT11HA_3_ for overexpression of TbGT11 fused to a C-terminal triple HA tag were fixed with 4% paraformaldehyde and permeabilized with 0.1% Triton X-100. Cells were co-stained with 0.3 μg/ml of mouse anti-HA antibody (kind gift of Dario Alessi, University of Dundee, UK) and rabbit anti-enolase serum (1:2000, kindly provided by Paul Michels, Catholic University of Louvain, Belgium), rabbit anti-BiP serum (1:2000, kind gift of Jay Bangs, University of Wisconsin-Madison, WI), rabbit polyclonal anti-trypanopain (1:300, kind gift of Jay Bangs, University of Wisconsin-Madison), or anti-Golgi reassembly stacking protein antisera (1:500, kindly provided by Graham Warren, MFPL, Austria) followed by incubation with Alexa Fluor 594-conjugated goat anti-mouse IgG and Alexa Fluor 488-conjugated goat anti-rabbit IgG secondary antibodies (Invitrogen). Coverslips were mounted in ProLong Gold mounting medium containing DAPI (Invitrogen) and imaged under a Delta Vision Core deconvolution microscope (Applied Precision, Inc.).

## RESULTS

### 

#### 

##### Analysis of the TbGT11 Gene Product

A family of 21 genes encoding putative UDP-sugar-dependent GTs was previously found in the *T. brucei* genome (19). In the current study, one of these genes (Tb927.3.5660) was selected for functional analysis. It encodes for a 388-amino acid protein with a theoretical molecular mass of 49.5 kDa. Although a semi-quantitative RT-PCR analysis suggested that this gene is expressed in similar levels in both bloodstream and procyclic form parasites (19), SILAC-based quantitative proteomics data demonstrated that at the protein level, the expression is more than 40 times higher in the bloodstream form (35).

When analyzed by software for predicting transmembrane helices based on a hidden Markov model (36), the protein is predicted to be a type II transmembrane protein, a hallmark of Golgi apparatus glycosyltransferases (37). Proximal to the predicted transmembrane domain, the N-terminal cytoplasmic tail contains a dibasic motif, which functions as an ER exit signal in known Golgi resident glycosyltransferases (38). Moreover, the protein contains a D*X*D motif (39), another common feature found in glycosyltransferases, as well as three putative *N*-glycosylation sites.

The *T. brucei* strain that was used for the genome sequencing project (TREU929) is different from the one that was used in this study (Lister strain 427). Alignment of Tb927.3.5660 and its homologue Tb427.3.5660 revealed a very high similarity with only 8 single nucleotide polymorphisms. Two of them results in amino acid changes (strain 427 encodes for Leu-175 in place of Ile-175 and Val-347 instead of Met-347). The strain 427 gene and protein product will be referred to here as *TbGT11* and TbGT11, respectively.

##### Creation of Bloodstream-form TbGT11 Null and Conditional Null Mutants

**FIGURE 1.**
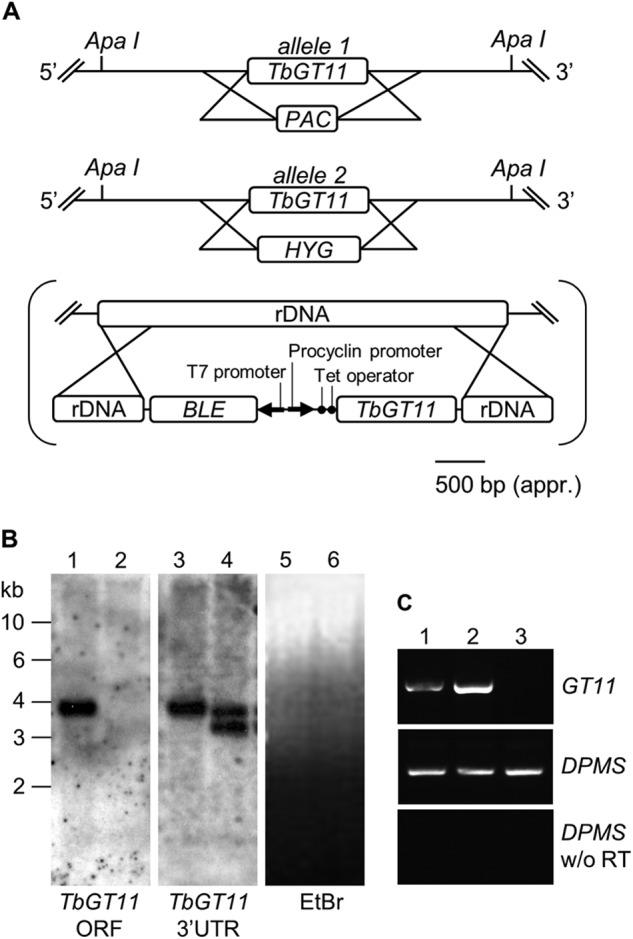
**Generation of a bloodstream-form *TbGT11* null and conditional mutant.** *A,* gene replacement strategy to create *TbGT11* null mutant cells and subsequent insertion of tetracycline-inducible ectopic copy, in *brackets*, to create a conditional null mutant. *B*, Southern blot of genomic DNA digested with ApaI from WT (*lanes 1*, *3*, and *5*) and *TbGT11* null mutant cells (*lanes 2*, *4*, and *6*). The blot was probed with a *TbGT11* ORF probe (*left hand panel*) and a *TbGT11* 3′ UTR probe (*middle panel*) and indicates the replacement of both alleles with drug resistance cassettes. Equal loading was verified by ethidium bromide staining (*right hand panel*). *C,* ethidium bromide-stained agarose gel of reverse transcription-PCR products from RNA extracted from WT cells (*lane 1*) and *TbGT11* conditional null mutants grown under permissive (*lane 2*) or non-permissive conditions (*lane 3*). The *upper panel* shows RT-PCR products using primers for *TbGT11*, the *middle panel* is a control using dolicholphosphate mannose synthase (*DPMS*) primers to show equal RNA input, and the *lower panel* is a control without reverse transcriptase.

Blast search of the *T. brucei* genome indicated that *TbGT11* is present as a single copy per haploid genome. Both alleles were replaced sequentially in the bloodstream-form parasite with *PAC* and *HYG* drug resistance cassettes by homologous recombination as summarized in Fig. 1*A*. Clones were selected on the relevant antibiotics and the generation of a *TbGT11* null mutant (Δ*TbGT11*::*PAC*/Δ*TbGT11*::*HYG*) was confirmed by Southern blot (Fig. 1*B*). A tetracycline-inducible ectopic copy of the *TbGT11* gene was introduced into the rDNA locus of a null mutant clone using the pLEW100 vector (27) and phleomycin selection (Fig. 1*A*, in *brackets*). The generation of this conditional null mutant (Δ*TbGT11*^Ti^/Δ*TbGT11*::*PAC*/Δ*TbGT11*::*HYG*) was confirmed by RT-PCR (Fig. 1*C*).

**FIGURE 2.**
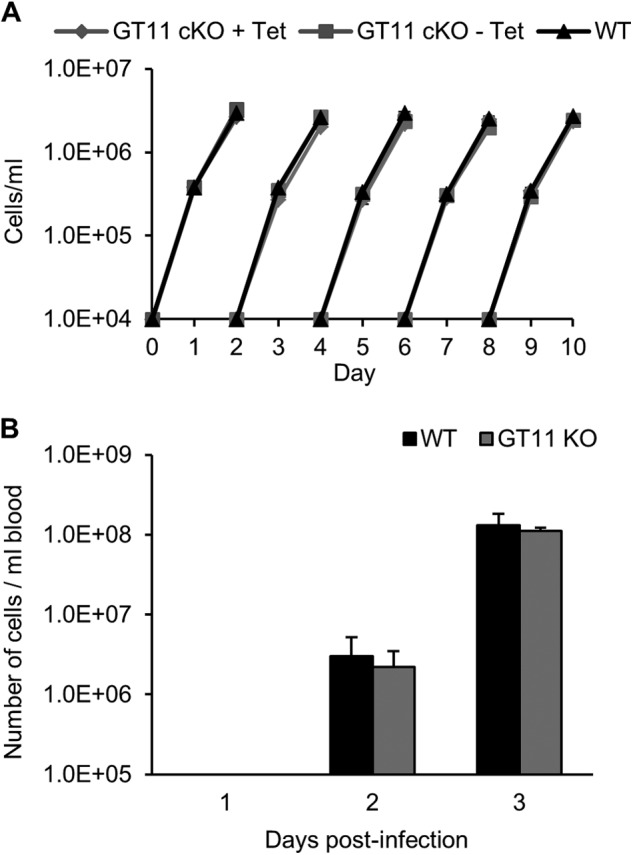
*A,* growth phenotypes of WT and *TbGT11* mutant cells. Cells were inoculated into culture, counted, and diluted to 1 × 10^4^ cells/ml with fresh media every 2 days. The growth characteristics for WT (black triangles), *TbGT11* conditional null cells under permissive (plus tetracycline, *gray diamonds*) and non-permissive (minus tetracycline, *gray squares*) conditions were indistinguishable. *B,* infectivity of WT and *TbGT11* null mutant bloodstream-form parasites to mice. Mice were infected with 1 × 10^5^ cells of WT (*black*) or *TbGT11* null mutants (*gray*) and parasite cell numbers were determined after 1, 2, and 3 days. No difference in infectivity was observed between WT and *TbGT11* null mutant cells.

The mutant cell lines had no noticeable differences in gross morphology, as judged by light microscopy (data not shown). Moreover, the *in vitro* growth rates of the *TbGT11* null mutant and its ability to infect mice were indistinguishable from their parental cell line (Fig. 2). From these data we conclude that *TbGT11* encodes a non-essential gene in *T. brucei* bloodstream-form cells.

##### Characterization of VSG from WT and TbGT11 Null Mutant Parasites

**FIGURE 3.**
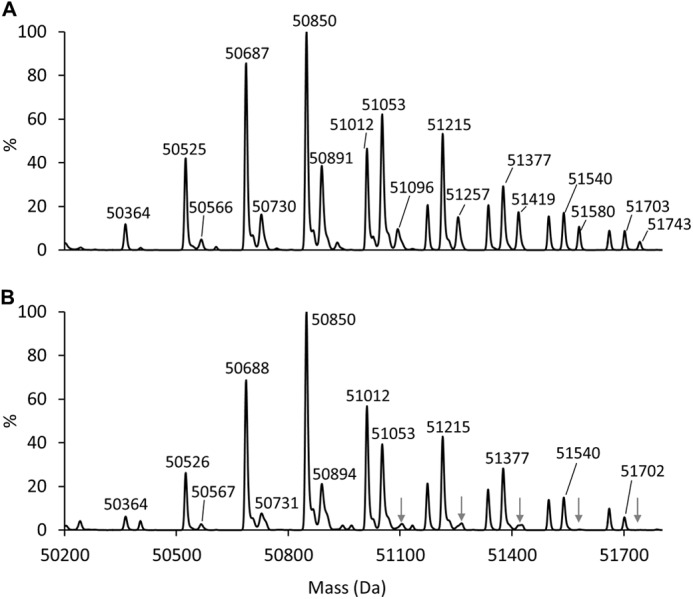
**Mass spectrometric analysis of intact sVSG221 from WT and *TbGT11* null mutant trypanosomes.** Samples of whole sVSG of WT (*A*) or *TbGT11* null mutant cells (*B*) were analyzed by ES-MS, and the spectra were deconvolved by maximum entropy. Significant differences in the sVSG glycoform patterns are indicated by *arrows* in *panel B*. The compositions of the various glycoforms are given in Table 1.

To perform glycosylation phenotyping of bloodstream-form *TbGT11* null mutants, VSG221 was purified in its soluble form. The VSG coat is released upon cell lysis by the action of endogenous GPI-specific phospholipase C (40). Intact sVSG glycoproteins from WT and mutant cells were analyzed by positive-ion ES-MS and the deconvolved mass spectra are depicted in Fig. 3. The typical glycoform pattern of wild-type sVSG221 (Fig. 3*A*) arises from its highly galactosylated GPI anchor (4) and its two *N*-glycans; triantennary oligomannose structures at Asn-428 (Man_5_-Man_9_GlcNAc_2_) and small biantennary structures ranging from Man_3_GlcNAc_2_ to Gal_2_GlcNAc_2_Man_3_GlcNAc_2_ at Asn-296 (5).

Although VSG glycoforms containing a total of four or five GlcNAc residues were present at similar levels in both genotypes, glycoforms with six GlcNAc residues were noticeably reduced in the *TbGT11* null mutant (see *arrows* in Fig. 3*B* and Table 1). Considering that the two *N*-glycan *N*-acetylchitobiose core structures account for four GlcNAc residues, this reduction in VSG glycoforms containing six GlcNAc residues suggests a reduction in the proportion of biantennary complex *N*-glycans. These data provided the first indication that the mutant cells cannot express complex *N*-glycans and that *TbGT11* is involved in their biosynthesis.

**TABLE 1:** **Isobaric glycoforms of sVSG221 identified by ES-MS** The molecular weights of different glycoforms of sVSG221 were calculated according to the indicated compositions (in parentheses is the theoretical mass of the assigned VSG composition). The relative abundances of those glycoforms observed in Fig. 3 for sVSG preparations from WT cells and *TbGT11* null mutant cells are indicated by: −, trace, +, ++, and +++ scores.

Molecular mass WT/*TbGT11* null mutant (theoretical)	Protein*^a^*	GlcN-Ino-cP*^b^*	EtNP	GlcNAc	Man + Gal	WT	*TbGT11* null mutant
*Da*							
50364/50364 (50356)	1	1	1	4	17	+*^c^*	Traces
50404/50404 (50397)	1	1	1	5	16	Traces	Traces
50525/50526 (50518)	1	1	1	4	18	++	++
50566/50567 (50559)	1	1	1	5	17	Traces	Traces
50607/NA (50600)	1	1	1	6	16	Traces	−
50687/50688 (50680)	1	1	1	4	19	+++	+++
50730/50731 (50721)	1	1	1	5	18	+	Traces
50850/50850 (50842)*^d^*	1	1	1	4	20	+++	+++
50891/50894 (50883)	1	1	1	5	19	++	+
50933/NA (50924)	1	1	1	6	18	Traces	−
51012/51012 (51004)	1	1	1	4	21	++	+++
51053/51053 (51045)	1	1	1	5	20	+++	++
51096/51103 (51086)	1	1	1	6	19	+	Traces
51174/51174 (51166)	1	1	1	4	22	+	+
51215/51215 (51207)	1	1	1	5	21	+++	++
51257/51261 (51248)	1	1	1	6	20	+	Traces
51337/51337 (51328)	1	1	1	4	23	+	+
51377/51377 (51369)	1	1	1	5	22	+	+
51419/51420 (51410)	1	1	1	6	21	+	Traces
51499/51499 (51490)	1	1	1	4	24	+	+
51540/51540 (51531)	1	1	1	5	23	+	+
51580/NA (51572)	1	1	1	6	22	+	−
51662/51661 (51652)	1	1	1	4	25	+	+
51703/51702 (51693)	1	1	1	5	24	+	Traces
51743/NA (51734)	1	1	1	6	23	Traces	−

*^a^* Protein *M*_r_ is based on the amino acid sequences of the VSG221 precursor (accession no. P26332) minus residues 1–27 (signal peptide) and 460–476 (GPI attachment signal peptide) and allows for four disulfide bonds (*M*_r_ = 46,284).

*^b^* Components specific to the GPI anchor and common to all glycoforms: GlcN-Ino-cP, glucosamine-α1–6-*myo*-inositol-1,2 cyclic phosphate; EtNP, ethanolamine phosphate.

*^c^* Maximum entropy deconvolved spectra are only semi-quantitative; an indication of the relative abundance of the isobaric glycoforms is given based on peak height.

*^d^* The most abundant glycoform of WT sVSG221 is expected to contain a GPI anchor of composition of Man_3_Gal_5_, a C-terminal *N*-linked glycan of Man_9_GlcNAc_2_, and an internal *N*-linked glycan of Man_3_GlcNAc_2_ (*i.e*. GlcNAc = 4, and Man = 20).

**FIGURE 4.**
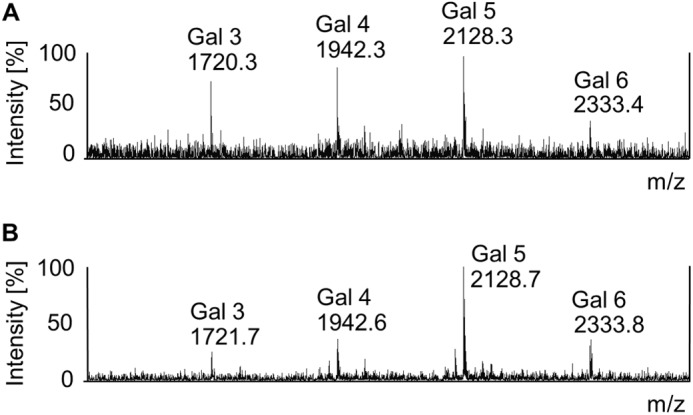
**MALDI-TOF mass spectra of permethylated GPI anchors isolated from WT and *TbGT11* null mutant sVSGs.** Permethylated GPI glycans of WT (*A*) or *TbGT11* null mutant (*B*) sVSGs were analyzed by MALDI-TOF MS. The annotated ions correspond to the permethylated GPI glycan core of Man_3_GlcNMe_3_-inositol with 3–6 side chain Gal residues, as indicated, together with their *m*/*z* values.

VSG GPI anchor dephosphorylation with aqueous hydrogen fluoride followed by permethylation and MALDI-TOF analysis showed no changes in the structure of the GPI anchor side chain (Fig. 4). This result shows that the intact VSG glycoform changes observed in Fig. 3 are due solely to changes in *N*-glycan structure.

##### N-Glycosylation Phenotype of Bloodstream-form TbGT11 Mutant Parasites

**FIGURE 5.**
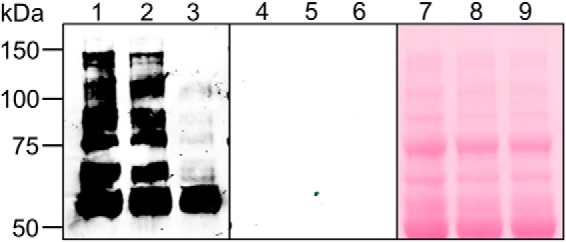
**Lectin blotting of total glycoproteins.** Western blot of total glycoproteins (depleted for VSG) of WT (*lanes 1*, *4*, and *7*), *TbGT11* conditional null mutants grown under permissive conditions (*lanes 2*, *5*, and *8*) and *TbGT11* conditional null mutants grown under non-permissive conditions (*lanes 3*, *6*, and *9*) incubated with ricin (*left-hand panel*). Binding specificity was confirmed by preincubating the lectin with 10 mg/ml of galactose and lactose (*middle panel*). Ponceau S staining shows equal loading and transfer between the lanes (*right-hand panel*).

To detect changes in the glycosylation of proteins other than VSG, total glycoproteins were extracted with SDS/urea from VSG-depleted trypanosome ghosts and analyzed by lectin blotting. As previously reported for WT *T. brucei* (19, 41), ricin (RCA-120), a lectin that predominantly binds to terminal β-galactose residues, showed strong binding to a series of glycoproteins running between 60 and 150 kDa (Fig. 5, *lane 1*). In contrast, ricin binding to glycoproteins extracted from the *TbGT11* conditional null mutants grown under non-permissive conditions was greatly reduced (Fig. 5, *lane 3*), indicating that TbGT11 might be play a role in the formation of hybrid and/or complex *N*-glycans. The phenotype was fully reversed in conditional null mutants grown under permissive conditions (in the presence of tetracycline) (Fig. 5, *lane 2*), confirming that the observed glycosylation defects are specifically due to the loss of TbGT11 expression.

**FIGURE 6.**
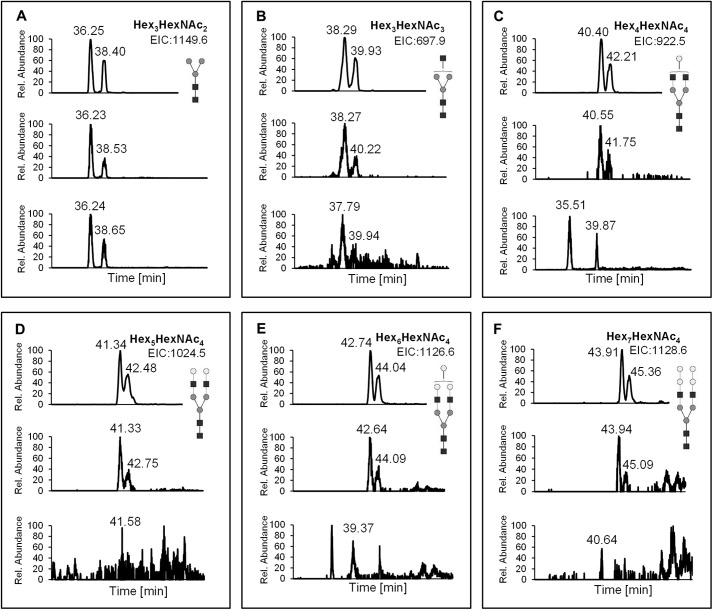
**Extracted ion chromatograms from LC-MS analysis of total *N*-glycans.** Total *N*-glycans of WT and *TbGT11* conditional null mutant cells grown under permissive or non-permissive conditions were enriched, permethylated, and analyzed by LC-MS. Shown are the extracted ion chromatograms of Hex_3_HexNAc_2_ (*A*), Hex_3_HexNAc_3_ (*B*), Hex_4_HexNAc_4_ (*C*), Hex_5_HexNAc_4_ (*D*), Hex_6_HexNAc_4_ (*E*), and Hex_7_HexNAc_4_ (*F*). In each box, the *upper panel* shows the chromatogram from WT cells, the *middle panel* represents the chromatogram from *TbGT11* conditional null mutant cells grown under permissive conditions, and the *lower panel* results from *TbGT11* conditional null mutant cells grown under non-permissive conditions.

To further advance the glycosylation phenotyping, the total *N*-glycan fraction from VSG-depleted cell ghosts was purified, permethylated, and analyzed by LC-MS. As shown in Fig. 6, extracted ion chromatograms revealed significant differences in the *N*-glycan pattern of WT (*upper panel*) and *TbGT11* conditional null mutant parasites grown under permissive conditions (*middle panel*) compared with *TbGT11* conditional null mutant parasites grown under non-permissive conditions (*lower panel*). Thus, whereas all three samples contain the conventional biantennary Manα1-3(Manα1-6)Manβ1-4GlcNAcβ1-4GlcNAc(Hex_3_HexNAc_2_) core as well as said structure extended by an additional GlcNAc residue (Hex_3_HexNAc_3_) (Fig. 6*A*, B), complex glycans with two GlcNAc residues attached to both 3- and 6-Man arms, *i.e.* GlcNAcβ1-2Manα1-3(GlcNAcβ1-2Manα1-6)Manβ1-4GlcNAcβ1-4GlcNAc and further elongated structures, were easily detected in WT cells and the TbGT11 conditional null mutant grown under permissive conditions but absent in the *TbGT11* null mutant grown under non-permissive conditions (Fig. 6, *C–F*). These data strongly suggest that TbGT11 has UDP-GlcNAc:glycoprotein GlcNAc transferase activity and is required for formation of biantennary complex *N*-glycans.

##### Analysis of VSG Pronase Glycopeptides of TbGT11 Null Mutant Cells Grown in the Presence and Absence of α-Mannosidase Inhibitors

**FIGURE 7.**
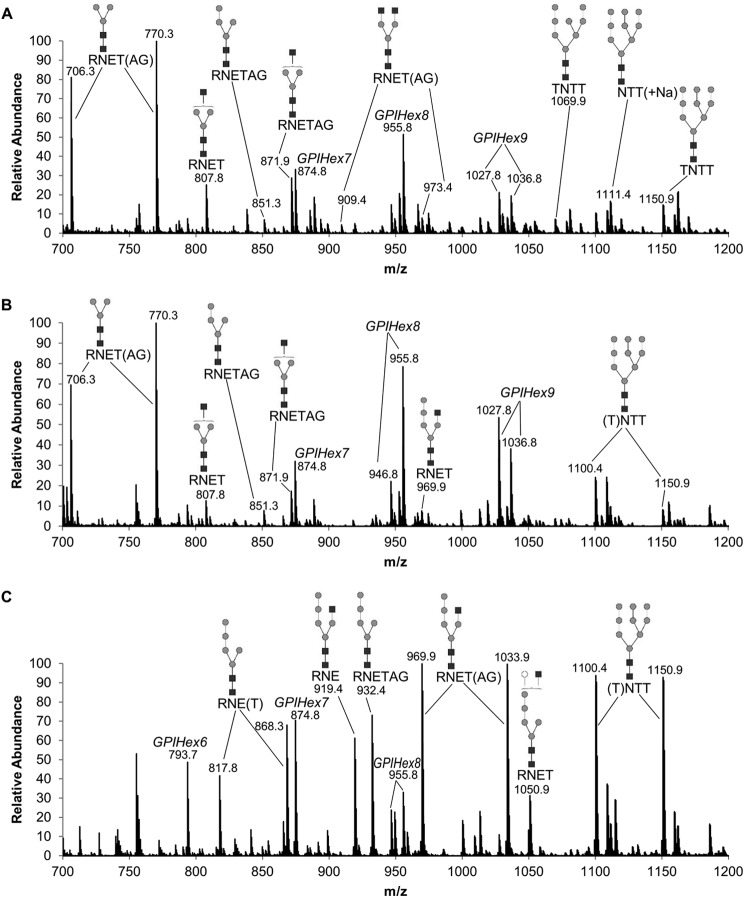
**ES-MS analysis of Pronase *N*-glycopeptides and GPI peptides.** Aliquots of sVSG221 were digested with Pronase and the resulting glycopeptides were enriched and analyzed by ES-MS in positive-ion mode. The identities of the ions from WT (*A*), *TbGT11* null mutant (*B*), and the *TbGT11* null mutant cells grown in the presence of α-mannosidase inhibitors (*C*) are indicated and were confirmed by MS/MS (data not shown). The *N*-linked glycopeptides appear as multiple ions due to incomplete Pronase digestion, *i.e.* the Asn-263 site is found as RNE-, RNET-, RNETAG-containing glycopeptides, and the Asn-428 site is found as NTT- and TNTT-containing glycopeptides.

Although the data described so far demonstrate that TbGT11 is responsible for GlcNAc transfer to the Manα1-3(Manα1-6)Manβ1-4GlcNAcβ1-4GlcNAc core, they contain no information as to whether the transfer is to the 3- or 6-arm. To answer this question, *TbGT11* null mutant cells were incubated with a mixture of cell-permeable α-mannosidase inhibitors (kifunensine, swainsonine, and 1-deoxymannojirimycin) for 72 h to inhibit *T. brucei* ER and Golgi α-mannosidase activity (15, 16). Extracted sVSGs from *TbGT11* null mutant cells treated with or without α-mannosidase inhibitors, as well as from WT cells, were then digested with Pronase. The resulting glycopeptides were enriched on graphitized carbon before analysis by ES-MS in a positive-ion mode. The identities of the detected ions were assigned based on their accurate mass and were confirmed by ES-MS/MS. The WT spectrum showed the typical range of known VSG glycopeptides (15, 16); *i.e.* small biantennary paucimannose and complex *N*-glycans attached to Asn-263 and triantennary oligomannose structures attached to Asn-428, as well as GPI-peptides (Fig. 7*A*). The *TbGT11* null mutant VSG Pronase glycopeptide spectrum showed the absence of complex *N*-glycan glycoforms, consistent with the previous data (Fig. 7*B*). The inhibition of α-mannosidase processing resulted in the replacement of Man_3_GlcNAc_2_-based structures by biantennary Man_5_GlcNAc_2_-based structures, as expected (Fig. 7*C*). However, the most important feature is the presence of the glycopeptide ions Hex_5_HexNAc_3_-RNE(TAG) (at *m*/*z* 919.4, 969.9, and 1033.9, respectively), as it demonstrates that biantennary Man_5_GlcNAc_2_ (Manα1-2Manα1-2Manα1-3(Manα1-6)Manβ1-4GlcNAcβ1-4GlcNAc) has been extended by a GlcNAc residue, presumably to the 6-arm by TbGnTII. Because this transfer to the 6-arm is clearly not influenced by the deletion of *TbGT11*, the enzymatic function of TbGT11 is most likely the transfer of β1–2-linked GlcNAc to the 3-arm; *i.e.* that of a GnTI enzyme.

##### In Vitro Functional Activity Assay

**FIGURE 8.**
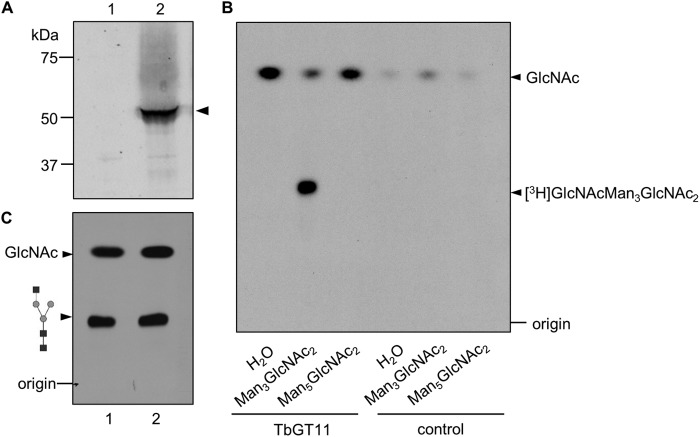
**TbGT11-HA_3_ expression and *in vitro* activity assay.** *A*, *Trypanosome* bloodstream-form cell lysates from WT cells (*lane 1*) or cells transfected with pLEW82-GT11-HA_3_ (*lane 2*) were separated by SDS-PAGE and analyzed by Western blotting with a rabbit anti-HA antibody. *B,* TLC autofluorography of *in vitro* reaction products. After incubation of TbGT11-HA_3_ attached to anti-HA-conjugated magnetic beads with UDP-[^3^H]GlcNAc as well as the acceptor substrates Man_3_GlcNAc_2_, Man_5_GlcNAc_2_, or no acceptor (H_2_O), reaction products were separated by TLC (*lanes 1–3*). As a negative control, anti-HA magnetic beads incubated with lysates from cells not expressing TbGT11-HA_3_ were used (*lane 4-6*). *C,* the obtained [^3^H]GlcNAcMan_3_GlcNAc_2_ reaction product was separated by TLC before and after α1–2,3 mannosidase treatment and visualized by fluorography.

To provide unambiguous evidence that TbGT11 is a glycosyltransferase and not simply required for the activity of another gene product, an *in vitro* assay for enzymatic activity was performed. Full-length TbGT11 fused to a C-terminal HA_3_ epitope tag was transfected into bloodstream-form *T. brucei* using a pLEW82 vector (27) and expression was confirmed by Western blot detection with an anti-HA antibody (Fig. 8*A*). Protein was immunoprecipitated with anti-HA magnetic beads and incubated with Manα1-3(Manα1-6)Manβ1-4GlcNAcβ1-4GlcNAc (oligomannose-3) or Manα1-2Manα1-2Manα1-3(Manα1-6)Manβ1-4GlcNAcβ1-4GlcNAc (oligomannose-5) as acceptor substrate and tritium-labeled UDP-[^3^H]GlcNAc as donor substrate. Reaction products were desalted and separated from unincorporated sugar nucleotides by mixed-bed ion exchange. Aliquots were analyzed by thin-layer chromatography (TLC) followed by autofluorography. As seen in Fig. 8*B*, oligomannose-3, but not oligomannose-5, is used as an acceptor substrate for TbGT11. No transfer was observed in a negative control without TbGT11-HA_3_. This clearly demonstrates that TbGT11 has glycosyltransferase activity and is able to transfer GlcNAc to biantennary Manα1-3(Manα1-6)Manβ1-4GlcNAcβ1-4GlcNAc core structures.

To determine whether GlcNAc is transferred to the 3- or 6-Man arms of oligomannose-3, tritium-labeled reaction products were treated with α1–2,3 mannosidase, a highly specific exoglycosidase that catalyzes the hydrolysis of the 3-Man arm. Samples were separated by TLC followed by fluorography. As shown in Fig. 8*C*, *R_f_* values of both samples were identical, consistent with the transferred GlcNAc residue being attached to the 3-Man arm and thereby impairing the cleavage by α1–3-mannosidase. This is in agreement with our previous data on the sVSG Pronase glycopeptides from α-mannosidase-treated *TbGT11* null mutants (Fig. 7*C*). Taken together, our data provide evidence that TbGT11 is responsible for GlcNAc transfer to the α1–3-linked d-mannopyranosyl residues of Manα1-3(Manα1-6)Manβ1-4GlcNAcβ1-4GlcNAc.

##### TbGT11 Is Localized in the Golgi Apparatus

**FIGURE 9.**
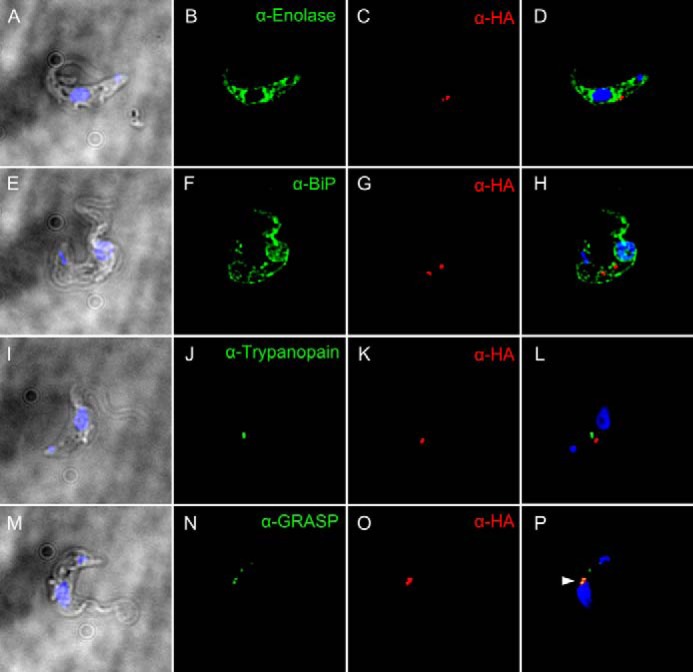
**Golgi localization of TbGT11.** Fixed and permeabilized bloodstream-form parasites expressing TbGT11-HA_3_ were co-stained with anti-HA antibodies to detect TbGT11 localization (*red*) and either anti-Enolase (*A–D*), anti-BiP (*E–H*), anti-trypanopain (*I–L*), or anti-Golgi reassembly stacking protein (*GRASP*) (*M–P*) to detect the cytosol, ER, lysosome, or Golgi apparatus, respectively (*green*). Cells were counterstained with DAPI (*blue*) to reveal nuclei and kinetoplasts. Merged DAPI/DIC images are presented on the *left* and merged three-channel fluorescence images are presented on the *right*. Prominent co-localization is indicated by an *arrowhead* (*P*, *yellow*). No staining of untransfected (non-epitope-tagged) cells was detected under the same conditions with anti-HA antibodies (data not shown).

Subcellular localization of HA-tagged TbGT11 was analyzed in *T. brucei* bloodstream-form parasites by immunofluorescence microscopy. Co-localization studies using antibodies against cell compartment-specific marker proteins and anti-HA antibodies are shown in Fig. 9. No staining of TbGT11 was detected in the cytosol (Fig. 9, *A–D*), the ER (Fig. 9, *E–H*), or the lysosome (Fig. 9, *I–L*), using enolase (42), BiP (43), and trypanopain (44) as markers, respectively. Instead, TbGT11 was clearly found to co-localize with Golgi reassembly stacking protein (GRASP) (45) (Fig. 9, *M–P*), consistent with being a Golgi-resident glycosyltransferase.

## DISCUSSION

The canonical modification of *N*-glycans by *GnTI* gene products to give rise to conventional complex *N*-glycan structures is widely considered as a hallmark of multicellular organisms, as both appeared in evolution at about the same time (46). Mammalian cell lines lacking a functional GnTI (*Mgat1*^−/−^) show normal growth in culture but mouse *Mgat1*-null mutants die at embryonic day 10 with severe multisystemic developmental abnormalities (11, 12). These developmental defects are consistent with the known roles of complex *N*-glycans in metazoan intercellular communication and signaling. On the other hand, *T. brucei* is a unicellular protozoan organism that also produces both conventional biantennary complex *N*-glycans and unique highly extended and branched poly-*N*-acetyllactosamine-containing *N*-glycan structures (5, 41, 47, 48). The precise role(s) of these complex structures, present in the bloodstream form of *T. brucei*, are less than clear. For example, poly-*N*-acetyllactosamine structures have been suggested to be involved in receptor-mediated endocytosis (49), but recent data on the glycosylation of the trypanosome transferrin receptor has questioned this function (50). Furthermore, the origins of these structures are also mysterious because canonical *GnTI* and *GnTII* genes, encoding the β1–2-GlcNAc transferases that normally initiate elaboration of the 3- and 6-arms, respectively, of the common Man_3_GlcNAc_2_ core are not obviously present in the *T. brucei* genome (19). In the present study *TbGT11* (Tb427.3.5660), which was predicted to encode a putative UDP-sugar dependent glycosyltransferase (19), has been shown to encode a Golgi apparatus enzyme that performs a GnTI-like function; *i.e.* the transfer of GlcNAc to the 3-arm of the Man_3_GlcNA_2_ *N*-glycan core via a UDP-*N*-acetylglucosaminyl:α1–3-d-mannoside-β1–2-*N*-acetylglucosaminyltransferase activity. We have therefore renamed TbGT11 to TbGnTI.

**FIGURE 10.**
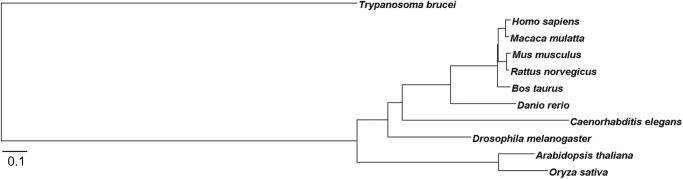
**Phylogenetic tree of GnTI amino acid sequences from different species.** Amino acid sequences were aligned using the COBALT constraint-based multiple alignment program. GnTI: *Homo sapiens* (AAH03575.1), *Macaca mulatta* (NP_001244759.1), *Mus musculus* (NP_001103620.1), *Rattus norvegicus* (AAH74010.1), *Bos taurus* (AAI51368.1), *Danio rerio* (NP_956970.1), *Caenorhabditis elegans* (AAD03022.1), *Drosophila melanogaster* (NP_525117.2), *Arabidopsis thaliana* (NP_849517.1), *Oryza sativa* (BAD28450.1). The *length* of the *horizontal lines* represents the evolutionary distance.

A multiple sequence alignment of TbGnTI and GnTI proteins of other species produced the phylogram shown in Fig. 10. The GnTIs of multicellular organisms are closely related and belong to the CAZy (carbohydrate-active enzymes) GT family 13 (51). The phylogram illustrates how divergent TbGnTI is from the canonical GnTI family. *TbGT11*, along with 20 other related *T. brucei* sequences, was identified by BLAST search using a human β3GnT5 query, a member of the CAZy GT 31 family (19). The trypanosome proteins all contain three motifs that are very similar to the (I/L)R*XX*WG, (F/Y)(V/L/M)*XXX*DXD, (ED)D(A/V)(Y/F)*X*G*X(*C/S) motifs conserved among members of the β3-glycosyltransferase family (52). Thus, whereas the only other characterized member of this trypanosome GT family is a β1–3-GlcNAc transferase (19), our present study reveals that a member of the β3-glycosyltransferase sequence family, TbGnTI, has β1–2-GlcNAc transferase activity. So far as we are aware, this is the first example of the repurposing of a β3-glycosyltransferase family member to catalyze the formation of another kind of glycosidic linkage. This might indicate that, apart from TbUGGT (24), trypanosomes have only one group of UDP-sugar-dependent GTs, evolved from a common ancestor β3-glycosyltransferase, to catalyze the variety of different linkages present in its diverse glycoconjugate repertoire (19). Consequently, the functional analysis of the other TbGT family members is an intriguing task for the future.

We were able to overexpress TbGnTI and show, by direct enzymatic assay, that whereas it recognizes Man_3_GlcNAc_2_, it cannot act on triantennary Man_5_GlcNAc_2_, which is the preferred acceptor substrate for vertebrate GnTI activities (46). This unusual specificity of TbGnTI is consistent with data presented in Refs. 16 and 17, which suggest that it is the presence of the α1–6-linked mannose residue transferred by the *ALG12* gene product in Man_5_GlcNAc_2_ that prevents TbGnTI from being able to transfer GlcNAc to the 3-arm and, therefore, makes *T. brucei* unable to make conventional hybrid *N*-glycans. In addition, the *N*-glycan profiles reported here for *TbGnTI* null mutant cells, grown with and without α-mannosidase inhibitors, demonstrate that the transfer of βGlcNAc to the 6-arm of the trimannosyl core is completely unaffected by the status of the 3-arm, which can be unsubstituted or substituted with mannobiose. The synthesis of these ”pseudohybrid“ *N*-glycans (53), which mirror the conventional hybrid structures found in multicellular organisms, shows clearly that TbGnTII can function without the prior action of TbGnTI.

The expression of TbGnTI is 40-fold higher in bloodstream-form compared with procyclic-form *T. brucei* at the protein level (35). This is consistent with the findings of Hwa and Khoo (54), who did not detect hybrid or complex *N*-glycans in wild-type procyclic forms. However, TbGnTI reaction products have been found in procyclic-form cells after the selection of ConA-resistant mutant clones (54, 55) and in procyclic-form *ALG12* and *ALG3* null mutants, indicating that TbGnTI activity can be evoked in procyclic-form trypanosomes in response to chemical or genetic challenges.

Finally, despite the significant changes in protein glycosylation brought about by deleting the *TbGnTI* (*TbGT11*) gene in bloodstream-form *T. brucei*, the *in vitro* growth rate and infectivity to mice of the null mutant were indistinguishable from wild type. This contrasts with the RNAi knockdown of TbSTT3A, the oligosaccharyltransferase responsible for transferring the biantennary Man_5_GlcNAc_2_ that is the precursor for paucimannose and complex *N*-glycans in *T. brucei*. In that case, the cells were viable in culture but not in mice (14). This clear difference in *in vivo* virulence between the *TbGnTI* null mutant and the *TbSTT3A* RNAi knockdown suggest that glycan extensions to the 6-arm alone in the pseudohybrid *N*-glycan structures that are created in the *TbGnTI* null might be able to compensate for those lost from the 3-arm. Whether the reverse is true must await identification of the gene(s) encoding TbGnTII activity.

## Footnotes

6 The abbreviations used are:

GPI: glycosylphosphatidylinositol
VSG: variant surface glycoprotein
sVSG: soluble-form VSG
GlcNAc: *N*-acetylglucosamine
Man: mannose
Glc: glucose
GT: glycosyltransferase
GnT: GlcNAc transferase
ER: endoplasmic reticulum
SILAC: stable isotope labeling with amino acids in cell culture
PAC: puromycin acetyltransferase
HYG: hygromycin phosphotransferase
TLCK: tosyllysine chloromethyl ketone hydrochloride
CAZy: carbohydrate-active enzymes
Dol-PP: dolichol pyrophosphate
bis-Tris: 2-[bis(2-hydroxyethyl)amino]-2-(hydroxymethyl)propane-1,3-diol.
